# A Pilot Study Providing Evidence for a Relationship between a Composite Lifestyle Score and Risk of Higher Carotid Intima-Media Thickness: Is There a Link to Oxidative Stress?

**DOI:** 10.1155/2018/4504079

**Published:** 2018-04-26

**Authors:** Neda Seyedsadjadi, Jade Berg, Ayse A. Bilgin, Ross Grant

**Affiliations:** ^1^School of Medical Sciences, Faculty of Medicine, University of New South Wales, Sydney, NSW 2052, Australia; ^2^Australasian Research Institute, Sydney Adventist Hospital, Sydney, NSW 2076, Australia; ^3^Department of Statistics, Macquarie University, Sydney, NSW 2109, Australia; ^4^Sydney Adventist Hospital Clinical School, University of Sydney, Sydney, NSW 2076, Australia

## Abstract

Lifestyle behaviours have been closely linked to the progressive cell damage associated with oxidative stress (OS) and the development of cardiovascular disease (CVD). Early detection of lifestyle-linked OS may therefore be useful in the early identification of prodromal disease. To test this hypothesis, this study assessed the relationship between a comprehensive redox balance lifestyle score (RBLS) and carotid intima-media thickness (CIMT), a recognized marker for CVD, and plasma biomarkers of OS. In a cross-sectional study design, 100 apparently healthy middle-aged participants were asked to complete a comprehensive lifestyle questionnaire, followed by DXA scanning, CIMT ultrasonography, and blood collection. The RBLS was composed of lifestyle components with pro- and antioxidant properties with a higher score indicative of lower oxidative activity. Multiple linear regression and logistic regression analysis were performed for statistical analysis. The RBLS was significantly associated with the risk for increased CIMT that was independent of conventional CVD risk factors (*χ*^2^(9) = 35.60, *P* ≤ 0.001). The adjusted model explained 42.4% of the variance in CIMT. Participants with RBLS below the median were at significantly increased risk of higher CIMT compared to participants with RBLS above the median (OR = 3.60, 95% CI: 1.19–10.88, *P* = 0.023). Significant associations were also observed between the RBLS, plasma total antioxidant capacity (TAC) (*r*(99) = 0.28, *P* = 0.006), hydroperoxide (HPX) (*r*_s_(99) = −0.28, *P* = 0.005), TAC/HPX ratio (*r*(98) = 0.41, *P* ≤ 0.001), *γ*-glutamyltransferase (*r*(97) = −0.23, *P* = 0.024), uric acid (*r*(98) = −0.20, *P* = 0.045), and inflammatory C-reactive protein (*r*_s_(97) = −0.25, *P* = 0.012) and interleukin-1*β* (*r*(97) = −0.21, *P* = 0.040). These findings highlight the importance of identifying the collective influence of lifestyle behaviours on OS activity and its potential to remodel the vascular endothelium.

## 1. Introduction

Cardiovascular diseases (CVDs) are the leading causes of death globally accounting for almost 30% of deaths worldwide [1]. The development of CVDs has a generally long prodromal period with clinical symptoms only becoming apparent after considerable damage, and remodelling has already occurred in the vascular endothelium. It has been shown that carotid intima-media thickness (CIMT) ultrasonography represents a sensitive and reliable method to detect the presence and progression of subclinical vascular changes that if left unchecked portend CVD [2]. However, in a recent study, more than half of CIMT variance was not explained by conventional cardiovascular risk factors such as dyslipidaemia [3]. Unfortunately, biochemical abnormalities such as high plasma total cholesterol are now recognized as downstream secondary pathologies, not as primary initiators of the disease process and therefore may not closely reflect actual disease, particularly in the early stages [4]. Thus, the identification of biomarkers for which the change more specifically represents the disease process and is therefore more indicative of the presence of tissue damage is needed.

It has been shown that biochemical changes associated with redox imbalance and oxidative stress are associated with the endothelial remodelling involved in atherosclerosis and increased CIMT [5, 6]. In addition, it has been reported that biomarkers of oxidative stress are significantly associated with the risk of atherosclerosis and CIMT even after controlling for conventional risk factors [7]. In other words, while generally not included in routine pathology measures such as plasma lipid profile, this chronic subclinical disease process may be detected via changes in oxidative stress biomarkers. This is consistent with the observation that oxidized low-density lipoprotein cholesterol (LDL-C) has been shown to be more atherogenic than native LDL-C [4]. Therefore, including a measure of oxidative stress in any risk assessment tool may add further value to the current set of conventional risk factors used for disease prediction and prognosis.

The role of unhealthy lifestyle behaviours as primary driving forces behind the development of CVDs has been well established [1]. Continuous exposure to unhealthy lifestyle behaviours, with high prooxidant and low antioxidant potentials, has been shown to significantly affect the redox balance causing oxidative stress and subsequent chronic cell damage [8]. Through years of chronic exposure, this damage accumulates and gradually drives the body toward a diseased phenotype [9]. Therefore, an early evaluation of the effect of lifestyle behaviours on the redox balance may be helpful in assessing whether an individual's lifestyle is driving their biochemistry toward either health or disease.

Much of the literature addressing the association between lifestyle behaviours, CVD risk, and oxidative stress has focused on the independent effects of individual lifestyle behaviours [10–14]. However, considering the potential for complex interactions between multiple lifestyle factors [15–17], a combination of several oxidative stress-related lifestyle factors, as a unified score, may be more strongly associated with health outcomes than any individual factor. While some studies have developed indices or scores that account for multiple lifestyle components, assessments of their effects on redox balance have been limited to lifestyle components such as dietary antioxidants/prooxidants, smoking, and medication use [18, 19]. In addition, their assessments have yielded conflicting results [18]. More importantly, none has investigated the association between an oxidative stress-related lifestyle score and a subclinical indicator of CVD, such as high CIMT. Hence, the associations between a redox balance lifestyle score, the risk for higher CIMT, and biomarkers of oxidative stress remain unclear. Therefore, in this study, we aimed to examine the association between a more comprehensive lifestyle score that included lifestyle factors previously shown to influence redox balance (i.e., redox balance lifestyle score (RBLS)) and risk for higher CIMT as a recognized subclinical marker for CVD. Secondarily, we investigated whether this RBLS also correlates with plasma biomarkers of oxidative stress and other conventional risk factors for atherosclerosis in otherwise healthy subjects. To the best of our knowledge, this is the first study to investigate these associations in an apparently healthy cohort.

## 2. Materials and Methods

### 2.1. Participants

In this cross-sectional study, 100 apparently healthy subjects (48 males and 52 females), aged between 40 and 75 years, were recruited at Sydney Adventist Hospital and the University of New South Wales campuses. After obtaining a written informed consent, participants were asked to complete a series of questionnaires for the assessment of their lifestyle behaviours. Participants were asked to recall their lifestyle behaviours over the past four months. All questionnaires (except dietary questionnaires) were completed online a maximum of two weeks before the blood collection and physiological assessments. Hard copies of dietary questionnaires were completed on the same day as the blood collection and physiological assessments. Blood collection, blood pressure measurement, and body scanning for visceral adipose tissue (VAT) fat mass analysis were all performed on the same day in a fasted state (about 12 hours). Ethical approval of the study was obtained from the Adventist HealthCare Limited Human Research Ethics Committee, Sydney Adventist Hospital, Australia (HREC number: 2013-022).

### 2.2. Biochemical Analysis

Plasma total antioxidant capacity and reactive oxygen species (in the form of hydroperoxides) were measured indirectly using the FORD (free oxygen radicals defence) and the FORT (free oxygen radical test) colorimetric assays (CR3000, Callegari Srl., Catellani Group, Parma, Italy), as previously described [20]. Plasma C-reactive protein (CRP) levels were quantified by immunoturbidimetric assay on a Roche/Hitachi cobas c system (Sydney Adventist Hospital pathology laboratory). Plasma tumour necrosis factor-*α* (TNF-*α*), interleukin-1*β* (IL-1*β*), and IL-6 levels were measured using the MILLIPLEX® MAP human high-sensitivity T-cell magnetic bead panel immunoassay (Merck KGaA, Darmstadt, Germany).

Measurements of fasting plasma glucose (FPG), total cholesterol (TC), high-density lipoprotein cholesterol (HDL-C), triglyceride (TG), uric acid (UA), and *γ*-glutamyltransferase (GGT) levels were conducted by the Sydney Adventist Hospital pathology laboratory on a Roche/Hitachi cobas c system using the enzymatic method. Plasma-glycated hemoglobin A1c (HbA1c) concentration was measured by ion-exchange high-performance liquid chromatography (HPLC) on the D-100 hemoglobin testing system (Bio-Rad Laboratories, Hercules, CA, USA).

Low-density lipoprotein cholesterol (LDL-C) levels were calculated by the Friedewald equation [21]. TyG index was calculated as the ln [fasting triglycerides (mmol/L) × fasting glucose (mmol/L)/2] [22].

### 2.3. Visceral Fat Mass Analysis

Dual-energy X-ray absorptiometry (DXA) method was used to measure VAT fat mass by a Lunar iDXA (GE Healthcare, Madison, WI, USA) with an automatic total-body scan mode and enCORE software (version 16, GE Healthcare, Madison, WI, USA). After being changed into a standard cloth gown, participants were correctly centred on the scanning table in a supine position and then were scanned by a trained operator according to the standard methods previously described [23].

### 2.4. Common Carotid Intima-Media Thickness (CIMT) Measurement

IMT measurements of the right common carotid artery (CCA) were obtained after placing the participant in a supine position and having them rotate their neck to the left. Orientation to the CCA was achieved by first using a transversal scanning view from the base of the neck to the carotid bifurcation. At approximately 1 cm below the bifurcation, the transducer was rotated to obtain a longitudinal image of the vessel. At least three representative measurements of CIMT were made in the far wall in the most thickened area of each vessel, while plaques were not included. The mean of these three measurements was then calculated and reported in the study. Optimal B-mode settings for gain, depth, and focal zone placement were adjusted to enhance arterial wall structures and image quality for each individual. IMT was measured by manually applying electronic callipers [24].

### 2.5. Questionnaires

For assessing dietary and alcohol intake, the validated 74-item Cancer Council Victoria Dietary Questionnaire for Epidemiological Studies Version 2 (DQES v2) [25] was used. The evaluation of caffeine intake, physical activity level, sitting time, depression, anxiety and stress, sleep quality, and sleep apnoea risk was conducted by using the validated Stanford questionnaire [26], International Physical Activity Questionnaire (IPAQ)—long version [27], Depression Anxiety Stress Scale-21 (DASS-21) questionnaire; Pittsburgh Sleep Quality Index (PSQI) [29], and Berlin questionnaires [30], respectively.

### 2.6. Statistical Analysis

The Shapiro-Wilk and Kolmogorov-Smirnov tests were applied to test the normality of the variables. After checking graphical displays and applying appropriate statistical rules, the following outliers were removed: one outlier was removed from the data for each of the variables stress score, anxiety score, sitting time, intakes of lycopene, *α*-carotene, *β*-carotene, polyunsaturated fatty acid (PUFA), iron, vitamin E and alcohol, plasma IL-6, TNF-*α*, TAC, and hydroperoxide. Therefore, *n* = 99 for each of these variables. In addition, one outlier was removed and one missing value was reported for the variable carotid intima-media thickness resulting in *n* = 98 for this variable. Two outliers were removed for the variables PSQI, depression score, intakes of caffeine and *β*-cryptoxanthin, plasma uric acid, and carotenoid resulting in *n* = 98 for each of these variables. Three outliers were removed for the variables vitamin C intake, VAT fat mass, plasma IL-1*β*, TG, CRP, GGT, and TG/HDL-C resulting in *n* = 97 for each of these variables. Correlations between variables were analyzed using Pearson's (*r*(*n*)) or Spearman's (*r*_s_(*n*)) correlation coefficients, as deemed appropriate. Multiple linear regression analysis was performed to examine the association between the RBLS and plasma levels of oxidative stress and inflammatory markers as well as other biochemical marker levels, after adjustment for age and gender. The Levene's test of equality was used to check the homogeneity of variances between groups. If normality tests for the variables or multiple linear regression models were significant, then an appropriate transformation of the data was performed so that normality assumptions were satisfied. Transformation techniques included base-10 log-transformed means, square roots, or reciprocal roots.

Logistic regression analysis was used to examine the association between the high CIMT risks and the RBLS. The median value was used to dichotomize the CIMT measure. Also, in order to allow a quantitative comparison of risks in persons at different ends of the exposure distribution, the RBLS was divided into two categories. All models were examined for collinearity among the independent variables. Linearity of any continuous independent variables and the logit transformation of the dependent variable were checked using the Box-Tidwell procedure. Whenever the distribution of a variable was significantly different from the normal distribution, we transformed that variable by using the power of 2 or the square root of the related independent variables was used. The results of the logistic regression models were expressed as adjusted odds ratios (ORs) and 95% CIs. All statistical analyses were performed using SPSS version 23 for Windows. *P* values less than 0.05 were considered statistically significant.

### 2.7. Redox Balance Lifestyle Score Definition

The redox balance lifestyle score (RBLS) was developed with reference to the previously validated oxidative balance score (OBS) [18]. OBS was comprised of lifestyle components with prooxidant and antioxidant properties including nutritional subcomponents, alcohol intake, smoking, and medication and supplement use. In order to provide a better estimation of the redox balance state, additional lifestyle behaviours with established evidence of their association with oxidative stress were included in the score calculation. These include intakes of transfatty acids [31] and caffeine [15]; psychological wellness [13]; sleeping behaviours [14]; physical activity [10]; and, for the first time, a measure of VAT fat mass, a well-known prooxidant risk factor for NCDs [32]. As all participants had been asked to stop taking medications or supplements at least two weeks prior to testing day, we did not take into account medication or supplement use.

The total score ranged from 4.54 to 9.05 points, with a higher score reflecting a better redox balance state (Table 1). All continuous score variables were divided into tertiles on the basis of the distribution of these variables among the participants of the study. The exception was for alcohol intake that was categorized as abstainer, one drink per day, and greater than 1 drink per day intakes, due to a significant number of abstainers in the cohort. The qualitative variable of apnoea risk was divided into two categories (low and high) based on the related questionnaire's scoring system. The smoking history cutoffs were defined by assigning one point to current smokers, two points to ever smokers, and three points to never smokers. Subjects who had values for components with antioxidant properties in the first, second, and third tertiles were assigned one, two, and three points, respectively. Subjects who had values for components with prooxidant properties in the first, second, and third tertiles were assigned three points, two points, and one point, respectively (Table 1).

In order to calculate the final RBLS, a raw score was first calculated for each person for each lifestyle component based on the scoring system described above. The raw score for each lifestyle component was then weighted by multiplying it by the (absolute) correlation coefficient of the association between the relevant component and the plasma TAC/HPX ratio (Table 1), a meaningful measure of redox balance [33]. Importantly, the potential for prooxidant effects due to excess antioxidant vitamin intake (e.g., vitamin E, vitamin C, or *β*-carotene) was accounted for by assigning a value of one to intakes higher than levels previously linked to oxidative activity [34, 35] for the related antioxidant. However, in this cohort, antioxidant vitamin intake values were below these pro-oxidant-linked values.

## 3. Results

Among a total of 100 subjects, 48 were males. Participant characteristics for individual components and their association with the redox balance lifestyle score (RBLS) are shown in Table 2. The participants' possible score can range between 4.54 (least healthy) and 9.05 (most healthy). The overall mean ± SD for the RBLS for the cohort was 6.69 ± 0.90. Statistically significant correlations were found between individual components and the overall RBLS as shown in Table 2. *β*-Carotene intake had the strongest association while transfatty acid and iron intakes had the weakest associations with the RBLS.

### 3.1. Association between the Redox Balance Lifestyle Score (RBLS) and Carotid Intima-Media Thickness (CIMT)

Table 3 shows the logistic regression analysis results for the association between redox balance lifestyle score (RBLS) and risk of higher CIMT. The logistic regression analysis showed that after adjustment for age and gender, there was a statistically significant (*χ*^2^(3) = 23.61, *P* ≤ 0.001) association between CIMT and the RBLS that explained 28.6% (Nagelkerke *R*^2^) of the variance in CIMT. Those with scores below the median (i.e., ≤6.68) were at significantly increased risk of higher CIMT compared to those with scores above the median (OR = 4.11, 95% CI: 1.57–10.75, *P* = 0.004).

Importantly, these associations remained significant after additional adjusting for conventional atherosclerosis risk factors including systolic and diastolic blood pressures, LDL-cholesterol, eGFR, FPG, and TG/HDL-C (*χ*^2^(9) = 35.60, *P* ≤ 0.001), where the adjusted model then explained 42.4% (Nagelkerke *R*^2^) of the variance in CIMT. After adjustment, those with the RBLS below the median (i.e., ≤6.68) were at a significantly increased risk of higher CIMT compared to participants with scores above the median (OR = 3.60, 95% CI: 1.19–10.88, *P* = 0.023).

### 3.2. Association between the Redox Balance Lifestyle Score (RBLS) and Oxidative Stress and Inflammatory Markers

Table 4 shows the associations between the RBLS and levels of plasma biomarkers of oxidative stress and inflammation. A statistically significant positive association was observed between the RBLS and total antioxidant capacity (TAC) (*r*(99) = 0.28, *P* = 0.006). Since the residuals for the linear regression model were not normally distributed when gender was included, the association between the RBLS and plasma TAC was analyzed separately for each gender. The results showed that after controlling for age, there was a statistically significant association between the RBLS and TAC in males (*t*(45) = 3.71, *P* = 0.001, *R*^2^ = 0.20) but not in females (*t*(48) = 0.98, *P* ≥ 0.05, *R*^2^ = −0.02). A statistically significant negative association was observed between the RBLS and plasma hydroperoxide levels (*r*_s_(99) = −0.28, *P* = 0.005). This association remained significant after the adjustment for age and gender (*t*(95) = −3.02, *P* = 0.003, *R*^2^ = 0.31).

There was a statistically significant negative association between the RBLS and the plasma oxidative stress-associated enzyme GGT (*r*(97) = −0.23, *P* = 0.024). This association remained significant after adjustment for age and gender (*t*(93) = −2.31, *P* = 0.023, *R*^2^ = 0.10).

The RBLS was significantly negatively associated with plasma uric acid (*r*(98) = −0.20, *P* = 0.045). This association remained significant after age and gender adjustment (*t*(94) = −2.43, *P* = 0.017, *R*^2^ = 0.30).

**Figure 1:**
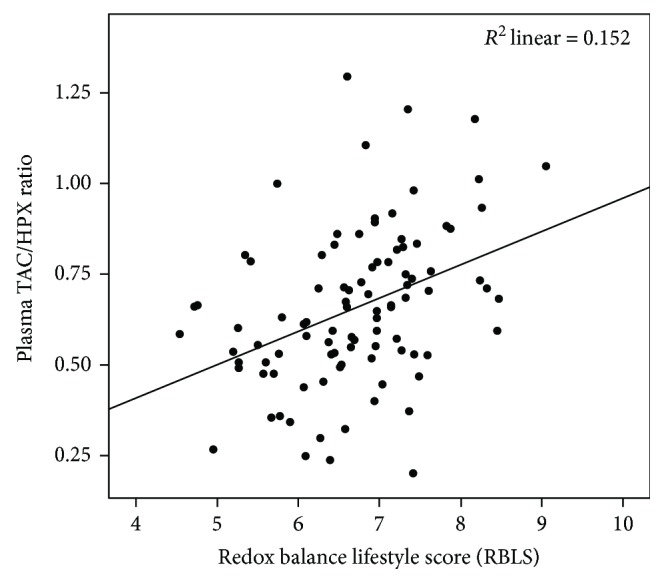
Association between the redox balance lifestyle score (RBLS) and plasma redox balance indicator of the TAC/HPX ratio.

As anticipated, the RBLS was statistically significantly associated with the TAC/HPX ratio (*r*(98) = 0.41, *P* ≤ 0.001), an index of redox balance (Figure 1). This association remained significant after adjustment for age and gender (*t*(94) = 4.40, *P* ≤ 0.001, *R*^2^ = 0.20).

There was a statistically significant negative association between the RBLS and the plasma inflammatory marker IL-1*β* (*r*(97) = −0.21, *P* = 0.04). This association did not remain significant after adjustment for age and gender (*t*(93) = −2.06, *P* ≥ 0.05, *R*^2^ = 0.05). The RBLS was also significantly associated with the acute-phase plasma inflammatory protein CRP (*r*_s_(97) = −0.25, *P* = 0.012). However, this association did not remain significant after age and gender adjustment (*t*(93) = −2.36, *P* ≥ 0.05, *R*^2^ = 0.04). No significant association was observed between the RBLS and plasma TNF-*α* (*r*(99) = −0.06, *P* ≥ 0.05) and IL-6 (*r*(98) = −0.07, *P* ≥ 0.05).

### 3.3. Association between the RBLS and Routine Pathology Biomarkers

A statistically significant negative association was observed between the RBLS and both plasma cholesterol (*r*(100) = −0.34, *P* ≤ 0.001) and LDL-C levels (*r*(100) = −0.32, *P* = 0.001). Both of these associations remained significant after adjustment for age and gender (*t*(96) = −3.63, *P* ≤ 0.001, *R*^2^ = 0.12 and *t*(96) = −3.42, *P* = 0.001, *R*^2^ = 0.11, resp.). There was no significant association between the RBLS and plasma HDL-C (*r*(100) = 0.12, *P* ≥ 0.05).

There was a statistically significant inverse association between the RBLS and plasma triglycerides (*r*_s_(97) = −0.32, *P* = 0.002). This association remained significant after adjusting for age and gender (*t*(93) = −3.81, *P* ≤ 0.001, *R*^2^ = 0.15).

A statistically significant inverse association was observed between the RBLS and both plasma glucose (*r*(100) = −0.30, *P* = 0.003) and HbA1c (*r*(95) = −0.23, *P* = 0.025). After adjustment for age and gender, the association between RBLS and plasma glucose remained significant (*t*(96) = −3.22, *P* = 0.002, *R*^2^ = 0.12). However, the association between RBLS and plasma HbA1c did not remain significant after adjustment for age and gender (*t*(91) = −2.39, *P* ≥ 0.05, *R*^2^ = 0.05).

Not surprisingly, a strong inverse association was observed between the RBLS and the plasma TG/HDL-C ratio (a measure of insulin resistance) (*r*(97) = −0.27, *P* = 0.008). This association remained significant after adjusting for age and gender (*t*(91) = −2.99, *P* = 0.004, *R*^2^ = 0.22).

Furthermore, a strong inverse association was observed between the RBLS and plasma TyG index (an additional measure of insulin resistance) (*r*(100) = −0.40, *P* ≤ 0.001). Since the residuals for the linear regression model were not normally distributed when gender was included, the association between the RBLS and plasma TyG index was analyzed separately for each gender. The results showed that after controlling for age, there was a statistically significant association between the RBLS and TyG index in both males (*t*(45) = −3.53, *P* ≤ 0.001, *R*^2^ = 0.18) and females (*t*(49) = −2.60, *P* = 0.012, *R*^2^ = 0.09).

## 4. Discussion

Oxidative stress (OS), defined as an imbalanced state between an organ's exposure to reactive oxygen species and the body's compensatory antioxidant defence [36], is a recognized player in the progressive cell damage involved in the development of CVD [4]. The specific impact of individual lifestyle components, with both pro- and antioxidant elements, on oxidative stress and the consequent development of several diseases including CVD have been a focus of attention in the literature for some time [10, 37–39]. However, as suggested by some investigators, it is not the individual but the cumulative action of all lifestyle factors working together that eventually affects the redox balance and determines an individual's risk of disease. In this context, some studies have reported associations between a composite lifestyle score and increased risk for several diseases [40, 41]. However, assessments of multiple lifestyle elements have been limited to a score comprising only a few pro- and antioxidant lifestyle factors with uncertain effect on oxidative stress biomarkers [18, 19]. Furthermore, no study has yet assessed the association between a composite oxidative stress-related lifestyle score and the subclinical vascular changes represented by CIMT. While one large study has reported an association between a seven-point cardiovascular health score and CIMT, its assessment was limited to only four lifestyle components with no reference to oxidative stress [42]. Therefore, in this study, we aimed to assess the association between a more comprehensive redox balance lifestyle score (RBLS) and CIMT. We also investigated the association between RBLS, oxidative stress and inflammatory biomarkers.

As expected, significant correlations were observed between individual lifestyle components and the overall RBLS (Table 2). All significant associations were in accordance with expected direction, with poorer lifestyle habits associated with increased oxidative stress.

In order to compare CIMT risks in subjects at different ends of the score distribution, the RBLS and CIMT values were divided into two groups using median values as the cutoffs. Logistic regression analysis showed that a lower RBLS was associated with a higher CIMT risk after adjusting for age and gender. More surprisingly, this association remained significant after further adjusting for conventional atherosclerosis risk factors including systolic and diastolic blood pressures, LDL-cholesterol, eGFR, FPG, and TG/HDL-C ratio. Subjects with scores below the median were 3.60 times more likely to exhibit thicker CIMT than subjects with scores above the median. As the oxidative modification hypothesis of atherosclerosis suggests, it is the accumulation of the oxidized LDL-C, not the native state of LDL-C, that is atherogenic [4]. This is consistent with our finding that the association between the RBLS and CIMT was independent of the conventional atherosclerosis risk factors such as LDL-C. While others have reported associations between CIMT, individual lifestyle behaviours, and a seven-point cardiovascular health score [42–45], to the best of our knowledge, this is the first study to report the association between a composite redox balance lifestyle score and CIMT.

We observed a significant positive association between the RBLS and total antioxidant capacity (TAC) that was independent of age in males but not in females. Consistent with this finding, in one study, the combination of both physical activity and adherence to a healthy diet was associated with high plasma TAC values [17]. We also observed a significant negative association between the RBLS and plasma lipid hydroperoxide levels, which was independent of age and gender. Similar to our finding, Lakkur et al. showed that their suggested oxidative balance score (OBS) was inversely associated with lipid peroxidation biomarker of F2-isoprostanes [18].

As anticipated, our results showed a strong positive association between the RBLS and the TAC/HPX ratio, an effective index of redox balance, which was independent of age and gender. This supports the view that various lifestyle behaviours may synergistically shift the body's biochemistry toward an imbalanced redox state and consequent oxidative stress. While no study has yet reported associations between TAC/HPX ratio and a comprehensive lifestyle score, our lab has previously reported on the correlation between individual lifestyle components and the TAC/HPX ratio, a redox balance index, using the same cohort [33].

Elevated plasma GGT levels have been suggested as an early biomarker of oxidative stress [46] and more importantly are associated with CIMT [47]. Consistent with this, we found a significant negative association between the RBLS and plasma GGT levels that was independent of age and gender. In agreement with this finding, in one study, a negative association was reported between an oxidative balance score (comprising BMI, nutrition, physical activity, and smoking components) and plasma GGT levels [19].

Elevated plasma uric acid levels have been shown to possess prooxidant properties in CVD [48] and also to be an independent risk factor for increased CIMT [49]. We were therefore not surprised to find a significant negative association between the RBLS and plasma uric acid levels. Consistent with this finding, one study has previously reported significant associations between plasma uric acid levels and individual lifestyle components such as BMI, nutrition, and physical activity [50].

It is recognized that oxidative stress and inflammation are interrelated. Oxidative stress can trigger an inflammatory response, which, in turn, can be a direct inducer of oxidative stress [51, 52]. Consistent with this, we observed a significant negative association between the RBLS and the plasma inflammatory marker IL-1*β* and also a significant negative association between the RBLS and the acute-phase plasma inflammatory protein CRP, which were both dependent on age and gender. Similarly, Lakkur et al. showed a significant association between an oxidative balance score (OBS) and plasma CRP [53].

Our results showed that the RBLS was associated not only with oxidative stress and inflammatory biomarkers but also with routine pathology lipid biomarkers. Significant negative associations were observed between the RBLS and plasma levels of total cholesterol and LDL-C that were both independent of age and gender. This finding is consistent with the results of a population-based study by Lakkur et al. in which OBS was significantly associated with plasma total cholesterol and LDL-C [53]. Consistent with Lakkur et al. [53], our data also showed no significant association between the RBLS and plasma HDL-C. We observed a strong negative association between the RBLS and plasma TG levels that was independent of age and gender. Similarly, in one study, a healthy lifestyle score (comprising BMI, nutrition, physical activity, and smoking components) was reported to be negatively associated with plasma TG in young Australian adults [54].

We observed a statistically significant negative association between the RBLS and plasma glucose, independent of age and gender, and HbA1c, which was dependent on age and gender. A number of other studies have shown a negative association between lifestyle scores (comprising components such as BMI, nutrition, and physical activity) and plasma glucose and HbA1c levels [54–56]. These results suggest an interrelationship between oxidative stress and plasma biomarker levels in response to lifestyle.

In further support of the role for oxidative stress in the development of CVD and related comorbidities, we observed strong negative and independent associations between the RBLS and surrogate markers of insulin resistance, triglyceride to HDL-C ratio (TG/HDL-C), and TyG index [22, 57]. Consistent with this finding, other studies have shown negative associations between healthy lifestyle scores (comprising components such as BMI, nutrition, and physical activity, smoking, social support, and sleep) and plasma levels of insulin [54, 55, 58].

In light of findings from a recent study in which more than half of the CIMT variance was not explained by conventional cardiovascular risk factors, additional contributors need to be identified [3]. Oxidative stress biomarkers have been previously shown to be significantly associated with the risk of atherosclerosis and increased CIMT after controlling for conventional risk factors of atherosclerosis [7]. As the RBLS in our study was associated with the risk of higher CIMT independent of conventional CVD risk factors and was also associated with oxidative stress biomarkers, it can be suggested that oxidative stress biomarkers may contribute to this role. Lifestyle factors with more prooxidant and less antioxidant properties can synergistically work together to shift the body's biochemistry toward a state of oxidative stress, which can cause subclinical changes in CIMT and gradually lead to the tissue remodelling associated with CVD.

While the conclusions in this study are robust, our study had some limitations. First, the cross-sectional design of the study does not allow confirmation of causality, but can generate hypotheses and thus stimulate future research. Also the relatively small number of subjects may decrease the sensitivity for identification of relationships with small effect sizes. Future longitudinal studies overcoming these limitations are required to verify the consistency of our observations. As lifestyle behaviour information was self-reported, measurement error and misclassification may occur. While validated questionnaires were applied to lessen such limitations, assessing exposures over a four-month period was another limitation of this study when long-term exposures may be more relevant to the development of atherosclerosis. Therefore, future studies overcoming these limitations are recommended.

## 5. Conclusion

In this study, for the first time, we showed significant associations between a composite redox balance lifestyle score (RBLS), risk for increased CIMT, and biomarkers of oxidative stress and inflammation in otherwise healthy subjects. Our findings provide further support for the role of multiple lifestyle factors influencing oxidative activity and the development of CVD. This suggests that monitoring oxidative activity may help with early identification of individuals at risk of CVD, allowing the institution of on-time, targeted, lifestyle-linked risk modifications.

## Figures and Tables

**Table 1:** RBLS's point assignment scheme.

Lifestyle component				Correlation coefficient with TAC/HPX ratio
*Energy storage (0.11–0.33 points)^*γ*^*				
VAT fat mass (g)	1 = high (3rd tertile)	2 = medium (2nd tertile)	3 = low (1st tertile)	−0.11^a^
*Nutritional factors (1.4–4.2 points)*				
PUFA intake	1 = high (3rd tertile)	2 = medium (2nd tertile)	3 = low (1st tertile)	0.14^a^
Trans-FA intake	1 = high (3rd tertile)	2 = medium (2nd tertile)	3 = low (1st tertile)	−0.05
Iron intake	1 = high (3rd tertile)	2 = medium (2nd tertile)	3 = low (1st tertile)	0.10
Vitamin E intake	1 = low (1st tertile) or higher than 270 mg^†^	2 = medium (2nd tertile)	3 = high (3rd tertile)	0.19^a^
Vitamin C intake	1 = low (1st tertile) or higher than 500 mg^†^	2 = medium (2nd tertile)	3 = high (3rd tertile)	0.09
*α*-Carotene intake	1 = low (1st tertile) or higher than 15 mg^†^	2 = medium (2nd tertile)	3 = high (3rd tertile)	0.29^∗^
*β*-Carotene intake	1 = low (1st tertile) or higher than 15 mg^†^	2 = medium (2nd tertile)	3 = high (3rd tertile)	0.19^a^
Lycopene intake	1 = low (1st tertile) or higher than 15 mg^†^	2 = medium (2nd tertile)	3 = high (3rd tertile)	0.04
Lutein and zeaxanthin intake	1 = low (1st tertile) or higher than 15 mg^†^	2 = medium (2nd tertile)	3 = high (3rd tertile)	0.17
*β*-Cryptoxanthine intake	1 = low (1st tertile) or higher than 15 mg^†^	2 = medium (2nd tertile)	3 = high (3rd tertile)	0.14
*Common social drug use (0.44–1.32 points)*				
Caffeine intake	1 = high (3rd tertile)	2 = medium (2nd tertile)	3 = low (1st tertile)	−0.28^a∗^
Alcohol intake	1 = high (>1 drink/day)	2 = medium (1 drink/day)	3 = low (None)	−0.14^a^
Smoking history	1 = current smoker	2 = previous smoker	3 = never smoker	−0.02^a^
*Physical activity (0.38–1.14 points)*				
Physical activity	1 = sedentary/low	2 = moderate	3 = high	0.17
Sitting time	1 = high (3rd tertile)	2 = medium (2nd tertile)	3 = low (1st tertile)	−0.21^a∗^
*Psychological factors (0.34–1.02 points)*				
Depression score	1 = high (3rd tertile)	2 = medium (2nd tertile)	3 = low (1st tertile)	−0.18
Stress score	1 = high (3rd tertile)	2 = medium (2nd tertile)	3 = low (1st tertile)	−0.09
Anxiety score	1 = high (3rd tertile)	2 = medium (2nd tertile)	3 = low (1st tertile)	0.07
*Sleep quality (0.52–1.56 points)*				
PSQI	1 = high (3rd tertile)	2 = medium (2nd tertile)	3 = low (1st tertile)	−0.25^∗^
Apnoea risk	1 = high		3 = low	0.27^a∗^

TAC: total antioxidant capacity; HPX: hydroperoxide; VAT: visceral adipose tissue; PUFA: polyunsaturated fatty acids; PSQI: Pittsburgh sleep quality index. Comparisons were made using Pearson's correlation unless otherwise stated; ^a^comparisons were made using Spearman's correlation; ^∗^*P* < 0.05; ^*γ*^points were calculated by multiplying the point value of each lifestyle component by the (absolute) correlation coefficient of the association between the relevant component and plasma redox balance index of TAC/HPX ratio; ^†^the level previously linked to oxidative activity [34, 35].

**Table 2:** Participant characteristics for individual components of the RBLS and their association with the RBLS.

Characteristic	*n*	Mean ± SD	Correlation coefficient with RBLS
Lifestyle score (RBLS)	100	6.69 ± 0.90	—
Age	100	55.98 ± 8.83	−0.09
VAT fat mass (g)	97	953.12 ± 798.26	−0.27^∗^
PUFA intake (g/day)	99	11.5 ± 5.65	0.12
Trans FA intake (g/day)	100	0.38 ± 0.31	−0.05
Iron intake (mg/day)	99	12.13 ± 4.44	−0.05
Vitamin E intake (mg/day)	99	7.09 ± 2.83	0.32^∗^
Vitamin C intake (mg/day)	97	105.54 ± 46.80	0.37^∗^
*α*-Carotene intake (*μ*g/day)	99	714.03 ± 418.73	0.48^∗^
*β*-Carotene intake (*μ*g/day)	99	3784.80 ± 1819.34	0.51^∗^
Lycopene intake (*μ*g/day)	99	5143.93 ± 2639.29	0.04
Lutein and zeaxanthin intake (*μ*g/day)	100	1011.88 ± 447.43	0.50^∗^
*β*-Cryptoxanthine intake (*μ*g/day)	98	236.27 ± 172.67	0.38^∗^
Caffeine intake (mg/day)	98	303.06 ± 320.63	−0.40^∗a^
Alcohol intake (g/day)	99	7.57 ± 10.72	−0.32^∗a^
*Smoking history*	100		−0.17^a^
Current smoker		0	
Ever smoker		74	
Never smoker		26	
*Physical activity (%)*	100		0.28^∗a^
Sedentary/low		20	
Moderate		43	
High		35	
Sitting time (min/day/week)	99	399.78 ± 154.34	−0.21^∗a^
Depression score	98	2.46 ± 3.01	−0.38^∗a^
Stress score	99	4.76 ± 3.25	−0.32^∗a^
Anxiety score	99	1.56 ± 1.76	−0.15^a^
PSQI	97	4.34 ± 2.95	−0.50^∗a^
*Apnoea risk (%)*	100		0.44^∗a^
High risk		20	
Low risk		80	

RBLS: redox balance lifestyle score; VAT: visceral adipose tissue; PUFA: polyunsaturated fatty acids; PSQI: Pittsburgh sleep quality index. Comparisons were made using Pearson's correlation unless otherwise stated; ^a^comparisons were made using Spearman's correlation; ^∗^*P* < 0.05.

**(a):** 

	Mean ± SD (*n* = 98)
CIMT (mm)	0.76 ± 0.18

**(b):** 

	OR (95% CI)	*P* value	*R* ^2^	*χ* ^2^
Model A	4.11 (1.57–10.75)	0.004	28.6%	23.61
Model B	3.60 (1.19–10.88)	0.023	42.4%	35.60

Model A: adjusted for age and gender; model B: adjusted for age, gender, systolic blood pressures, diastolic blood pressures, low-density lipoprotein cholesterol, estimated glomerular filtration rate (eGFR), fasting plasma glucose, and triglyceride/high-density lipoprotein cholesterol ratio; RBLS: redox balance lifestyle score.

**Table 4:** Correlation coefficients and *P* values for the associations between RBLS and plasma biomarkers of oxidative stress, inflammation, and other biochemical markers.

	Correlation coefficient	*P* value
Oxidative stress biomarkers		
TAC (mmol/L)	0.28	0.006
HPX (mmol/L)	−0.28^a^	0.005
GGT (U/L)	−0.23	0.024
Uric acid (mmol/L)	−0.20	0.045
TAC/HPX	0.41	≤0.001
*Inflammatory biomarkers*		
IL-1*β* (pmol/L)	−0.21	0.040
CRP (mg/L)	−0.25^a^	0.012
TNF-*α* (pmol/L)	−0.06	NS
IL-6 (pmol/L)	−0.10^a^	NS
*Other biochemical markers*		
TC (mmol/L)	−0.34	≤0.001
LDL-C (mmol/L)	−0.32	0.001
HDL-C (mmol/L)	0.12	NS
TG (mmol/L)	−0.32^a^	0.002
FPG (mmol/L)	−0.30	0.003
HbA1c (mmol/mol)	−0.23	0.025
TG/HDL	−0.27	0.008
TyG index	−0.40	≤0.001

RBLS: redox balance lifestyle score; TAC: total antioxidant capacity; HPX: hydroperoxide; GGT: *γ*-glutamyltransferase; IL-1*β*: interleukin-1*β*; CRP: C-reactive protein; TNF-*α*: tumour necrosis factor-*α*; TC: total cholesterol; LDL-C: low-density lipoprotein cholesterol; HDL-C: high-density lipoprotein cholesterol; TG: triglyceride; FPG: fasting plasma glucose; HbA1c: glycated hemoglobin A. Comparisons were made using Pearson's correlation unless otherwise stated; ^a^comparisons were made using Spearman's correlation.

## Abbreviations

CIMT: Carotid intima-media thickness
CRP: C-reactive protein
CVD: Cardiovascular disease
DXA: Dual-energy X-ray absorptiometry
FPG: Fasting plasma glucose
GGT: *γ*-Glutamyltransferase
HbA1c: Glycated hemoglobin A1c
HDL-C: High-density lipoprotein cholesterol
HPX: Hydroperoxide
IL-1*β*: Interleukin-1*β*
LDL-C: Low-density lipoprotein cholesterol
NCDs: Noncommunicable disease
OS: Oxidative stress
PSQI: Pittsburgh sleep quality index
PUFA: Polyunsaturated fatty acid
RBLS: Redox balance lifestyle score
TAC: Total antioxidant capacity
TC: Total cholesterol
TG: Triglyceride
TNF-*α*: Tumour necrosis factor-*α*
UA: Uric acid
VAT: Visceral adipose tissue.

## References

[1] World Health Organization (2017). *Cardiovascular Diseases (CVDs) [Fact Sheet]*.

[2] Onut R., Balanescu A. P. S., Constantinescu D., Calmac L., Marinescu M., Dorobantu P. M. (2012). Imaging atherosclerosis by carotid intima-media thickness *in vivo*: how to, where and in whom?. *Maedica*.

[3] Santos I. S., Alencar A. P., Rundek T. (2015). Low impact of traditional risk factors on carotid intima-media thickness: the ELSA-brasil cohort. *Arteriosclerosis, Thrombosis, and Vascular Biology*.

[4] Huang H., Mai W., Liu D., Hao Y., Tao J., Dong Y. (2008). The oxidation ratio of LDL: a predictor for coronary artery disease. *Disease Markers*.

[5] Osorio J. M., Ferreyra C., Pérez A., Moreno J. M., Osuna A. (2009). Prediabetic states, subclinical atheromatosis, and oxidative stress in renal transplant patients. *Transplantation Proceedings*.

[6] Ari E., Kaya Y., Demir H. (2011). Oxidative DNA damage correlates with carotid artery atherosclerosis in hemodialysis patients. *Hemodialysis International*.

[7] Yoon J. H., Kim J. Y., Park J. K., Ko S. B. (2015). Oxidative damage markers are significantly associated with the carotid artery intima-media thickness after controlling for conventional risk factors of atherosclerosis in men. *PLoS One*.

[8] Dato S., Crocco P., D’Aquila P. (2013). Exploring the role of genetic variability and lifestyle in oxidative stress response for healthy aging and longevity. *International Journal of Molecular Sciences*.

[9] Kvaavik E., Batty G. D., Ursin G., Huxley R., Gale C. R. (2010). Influence of individual and combined health behaviors on total and cause-specific mortality in men and women: the United Kingdom Health and Lifestyle Survey. *Archives of Internal Medicine*.

[10] Sallam N., Laher I. (2016). Exercise modulates oxidative stress and inflammation in aging and cardiovascular diseases. *Oxidative Medicine and Cellular Longevity*.

[11] Harasym J., Oledzki R. (2014). Effect of fruit and vegetable antioxidants on total antioxidant capacity of blood plasma. *Nutrition*.

[12] Schrieks I. C., van den Berg R., Sierksma A., Beulens J. W. J., Vaes W. H. J., Hendriks N. F. J. (2013). Effect of red wine consumption on biomarkers of oxidative stress. *Alcohol and Alcoholism*.

[13] Maes M., Mihaylova I., Kubera M., Uytterhoeven M., Vrydags N., Bosmans E. (2009). Increased 8-hydroxy-deoxyguanosine, a marker of oxidative damage to DNA, in major depression and myalgic encephalomyelitis/chronic fatigue syndrome. *Neuro Endocrinology Letters*.

[14] Singh R., Kiloung J., Singh S., Sharma D. (2008). Effect of paradoxical sleep deprivation on oxidative stress parameters in brain regions of adult and old rats. *Biogerontology*.

[15] Olcina G. J., Timón R., Muñoz D., Maynar J. I., Caballero M. J., Maynar M. (2008). Caffeine ingestion effects on oxidative stress in a steady-state test at 75% VO_2_ max. *Science & Sports*.

[16] Yang C. L., Chen C. H. (2018). Effectiveness of aerobic gymnastic exercise on stress, fatigue, and sleep quality during postpartum: a pilot randomized controlled trial. *International Journal of Nursing Studies*.

[17] Panagiotakos D. B., Kavouras S. A., Pitsavos C. (2011). Physical activity and adherence to Mediterranean diet increase total antioxidant capacity: the ATTICA study. *Cardiology Research and Practice*.

[18] Lakkur S., Bostick R. M., Roblin D. (2014). Oxidative balance score and oxidative stress biomarkers in a study of Whites, African Americans, and African immigrants. *Biomarkers*.

[19] Cho A. R., Kwon Y. J., Lim H. J. (2017). Oxidative balance score and serum *γ*-glutamyltransferase level among Korean adults: a nationwide population-based study. *European Journal of Nutrition*.

[20] Lewis N. A., Newell J., Burden R., Howatson G., Pedlar C. R. (2016). Critical difference and biological variation in biomarkers of oxidative stress and nutritional status in athletes. *PLoS One*.

[21] Friedewald W. T., Levy R. I., Fredrickson D. S. (1972). Estimation of the concentration of low-density lipoprotein cholesterol in plasma, without use of the preparative ultracentrifuge. *Clinical Chemistry*.

[22] Simental-Mendía L. E., Rodríguez-Morán M., Guerrero-Romero F. (2008). The product of fasting glucose and triglycerides as surrogate for identifying insulin resistance in apparently healthy subjects. *Metabolic Syndrome and Related Disorders*.

[23] Seyed-Sadjadi N., Berg J., Bilgin A. A., Grant R. (2017). Visceral fat mass: is it the link between uric acid and diabetes risk?. *Lipids in Health and Disease*.

[24] Stein J. H., Korcarz C. E., Hurst R. T. (2008). Use of carotid ultrasound to identify subclinical vascular disease and evaluate cardiovascular disease risk: a consensus statement from the American Society of Echocardiography Carotid Intima-Media Thickness Task Force endorsed by the Society for Vascular Medicine. *Journal of the American Society of Echocardiography*.

[25] Ireland P. J. D., Giles G., O’Dea K. (1994). Development of the Melbourne FFQ: a food frequency questionnaire for use in an Australian prospective study involving an ethnically diverse cohort. *Asia Pacific Journal of Clinical Nutrition*.

[26] Nova P., Hernandez B., Ptolemy A. S., Zeitzer J. M. (2012). Modeling caffeine concentrations with the Stanford Caffeine Questionnaire: preliminary evidence for an interaction of chronotype with the effects of caffeine on sleep. *Sleep Medicine*.

[27] Craig C. L., Marshall A. L., Sjöström M. (2003). International physical activity questionnaire: 12-country reliability and validity. *Medicine & Science in Sports & Exercise*.

[28] Lovibond S. H., Lovibond P. F. (1995). *Psychology Foundation of Australia . Manual for the Depression Anxiety Stress Scales*.

[29] Buysse D. J., Reynolds Iii C. F., Monk T. H., Berman S. R., Kupfer D. J. (1989). The Pittsburgh sleep quality index: a new instrument for psychiatric practice and research. *Psychiatry Research*.

[30] Netzer N. C., Stoohs R. A., Netzer C. M., Clark K., Strohl K. P. (1999). Using the Berlin questionnaire to identify patients at risk for the sleep apnea syndrome. *Annals of Internal Medicine*.

[31] Anderson C., Milne G. L., Sandler D. P., Nichols H. B. (2016). Oxidative stress in relation to diet and physical activity among premenopausal women. *British Journal of Nutrition*.

[32] Fujita K., Nishizawa H., Funahashi T., Shimomura I., Shimabukuro M. (2006). Systemic oxidative stress is associated with visceral fat accumulation and the metabolic syndrome. *Circulation Journal*.

[33] Seyedsadjadi N., Berg J., Bilgin A. A., Tung C., Grant R. (2017). Significant relationships between a simple marker of redox balance and lifestyle behaviours; relevance to the Framingham risk score. *PLoS One*.

[34] Huang H. Y., Helzlsouer K. J., Appel L. J. (2000). The effects of vitamin C and vitamin E on oxidative DNA damage: results from a randomized controlled trial. *Cancer Epidemiology Biomarkers & Prevention*.

[35] Astley S. B., Hughes D. A., Wright A. J. A., Elliott R. M., Southon S. (2004). DNA damage and susceptibility to oxidative damage in lymphocytes: effects of carotenoids *in vitro* and *in vivo*. *British Journal of Nutrition*.

[36] Sies H. (1991). Oxidative stress: from basic research to clinical application. *The American Journal of Medicine*.

[37] Eisele H. J., Markart P., Schulz R. (2015). Obstructive sleep apnea, oxidative stress, and cardiovascular disease: evidence from human studies. *Oxidative medicine and cellular longevity*.

[38] Bernhard D., Wang X. L. (2007). Smoking, oxidative stress and cardiovascular diseases—do anti-oxidative therapies fail?. *Current Medicinal Chemistry*.

[39] Monguchi T., Hara T., Hasokawa M. (2017). Excessive intake of trans fatty acid accelerates atherosclerosis through promoting inflammation and oxidative stress in a mouse model of hyperlipidemia. *Journal of Cardiology*.

[40] Kong S. Y., Goodman M., Judd S., Bostick R. M., Flanders W. D., McClellan W. (2015). Oxidative balance score as predictor of all-cause, cancer, and noncancer mortality in a biracial US cohort. *Annals of Epidemiology*.

[41] Ilori T. O., Wang X., Huang M. (2017). Oxidative balance score and the risk of end-stage renal disease and cardiovascular disease. *American Journal of Nephrology*.

[42] Giannini C., Diesse L., D'Adamo E. (2014). Influence of the Mediterranean diet on carotid intima–media thickness in hypercholesterolaemic children: a 12-month intervention study. *Nutrition, Metabolism, & Cardiovascular Diseases*.

[43] Kawase Ishihara K., Kokubo Y., Yokota C. (2015). Effect of plasma fibrinogen, high-sensitive C-reactive protein, and cigarette smoking on carotid atherosclerosis: the Suita study. *Journal of Stroke & Cerebrovascular Diseases*.

[44] Zyriax B. C., Lau K., Klähn T., Boeing H., Völzke H., Windler E. (2010). Association between alcohol consumption and carotid intima–media thickness in a healthy population: data of the STRATEGY study (stress, atherosclerosis and ECG study). *European Journal of Clinical Nutrition*.

[45] Oikonen M., Laitinen T. T., Magnussen C. G. (2013). Ideal cardiovascular health in young adult populations from the United States, Finland, and Australia and its association with cIMT: the International Childhood Cardiovascular Cohort Consortium. *Journal of the American Heart Association*.

[46] Lim J. S., Yang J. H., Chun B. Y., Kam S., Jacobs D. R., Lee D. H. (2004). Is serum *γ*-glutamyltransferase inversely associated with serum antioxidants as a marker of oxidative stress?. *Free Radical Biology & Medicine*.

[47] Eroglu S., Sade L. E., Polat E., Bozbas H., Ulus T., Muderrisoglu H. (2011). Association between serum gamma-glutamyltransferase activity and carotid intima-media thickness. *Angiology*.

[48] Sautin Y. Y., Johnson R. J. (2008). Uric acid: the oxidant-antioxidant paradox. *Nucleosides, Nucleotides and Nucleic Acids*.

[49] Takayama S., Kawamoto R., Kusunoki T., Abe M., Onji M. (2012). Uric acid is an independent risk factor for carotid atherosclerosis in a Japanese elderly population without metabolic syndrome. *Cardiovascular Diabetology*.

[50] Liu L., Lou S., Xu K., Meng Z., Zhang Q., Song K. (2013). Relationship between lifestyle choices and hyperuricemia in Chinese men and women. *Clinical Rheumatology*.

[51] Wassmann S., Stumpf M., Strehlow K. (2004). Interleukin-6 induces oxidative stress and endothelial dysfunction by overexpression of the angiotensin II type 1 receptor. *Circulation Research*.

[52] Brigelius-Flohé R., Friedrichs B., Maurer S., Schultz M., Streicher R. (1997). Interleukin-1-induced nuclear factor *κ*B activation is inhibited by overexpression of phospholipid hydroperoxide glutathione peroxidase in a human endothelial cell line. *Biochemical Journal*.

[53] Lakkur S., Judd S., Bostick R. M. (2015). Oxidative stress, inflammation, and markers of cardiovascular health. *Atherosclerosis*.

[54] Gall S. L., Jamrozik K., Blizzard L., Dwyer T., Venn A. (2009). Healthy lifestyles and cardiovascular risk profiles in young Australian adults: the childhood determinants of adult health study. *European Journal of Preventive Cardiology*.

[55] Bhupathiraju S. N., Lichtenstein A. H., Dawson-Hughes B., Tucker K. L. (2011). Adherence index based on the AHA 2006 diet and lifestyle recommendations is associated with select cardiovascular disease risk factors in older Puerto Ricans. *The Journal of Nutrition*.

[56] Maseli A., Aeschbacher S., Schoen T. (2017). Healthy lifestyle and blood pressure variability in young adults. *American Journal of Hypertension*.

[57] McLaughlin T., Reaven G., Abbasi F. (2005). Is there a simple way to identify insulin-resistant individuals at increased risk of cardiovascular disease?. *The American Journal of Cardiology*.

[58] Sotos-Prieto M., Bhupathiraju S. N., Falcón L. M., Gao X., Tucker K. L., Mattei J. (2015). A healthy lifestyle score is associated with cardiometabolic and neuroendocrine risk factors among Puerto Rican adults. *The Journal of Nutrition*.

